# Social identification and risk dynamics: How perceptions of (inter)personal and collective risk impact the adoption of COVID‐19 preventative behaviors

**DOI:** 10.1111/risa.14155

**Published:** 2023-05-03

**Authors:** Mark Atkinson, Fergus Neville, Evangelos Ntontis, Stephen Reicher

**Affiliations:** ^1^ School of Management University of St Andrews St Andrews UK; ^2^ School of Psychology and Neuroscience University of St Andrews St Andrews UK; ^3^ School of Psychology & Counselling The Open University Milton Keynes UK

**Keywords:** collective risk, COVID‐19, pandemic, personal risk, preventative measures

## Abstract

Public adoption of preventative behaviors to reduce the transmission of COVID‐19 is crucial to managing the pandemic, and so it is vital to determine what factors influence the uptake of those behaviors. Previous studies have identified COVID‐19 risk perceptions as a key factor, but this work has typically been limited both in assuming that risk means risk to the personal self, and in being reliant on self‐reported data. Drawing on the social identity approach, we conducted two online studies in which we investigated the effects of two different types of risk on preventative measure taking: risk to the personal self and risk to the collective self (i.e., members of a group with which one identifies). Both studies involved behavioral measures using innovative interactive tasks. In Study 1 (*n* = 199; data collected 27 May 2021), we investigated the effects of (inter)personal and collective risk on physical distancing. In Study 2 (*n* = 553; data collected 20 September 2021), we investigated the effects of (inter)personal and collective risk on the speed at which tests are booked as COVID‐19 symptoms develop. In both studies, we find that perceptions of collective risk, but not perceptions of (inter)personal risk, influence the extent to which preventative measures are adopted. We discuss the implications both conceptually (as they relate to both the conceptualization of risk and social identity processes) and also practically (in terms of the implications for public health communications).

## 1 INTRODUCTION

One key lesson from the COVID‐19 pandemic has been the importance of behavior in determining the spread of infection. Widespread public adherence to preventative measures is a crucial component in the management of COVID‐19. Physical distancing,^1^ getting tested in the event of developing symptoms, and self‐isolating while infectious, for example, all play a role in reducing the spread of the virus, allowing schools, businesses, public transport, and other public spaces to remain open and safe. The ECDC (European Centre for Disease Prevention and Control) note that behavioral interventions “have played a critical role in reducing transmission rates and the impact of COVID‐19” (ECDC, 2021).

Identifying the factors which determine the extent to which members of the public adopt such preventative measures is therefore vital. It has implications for how they are promoted by Government and health authorities and, more specifically, how they should be communicated to the public.

Accordingly, there is a large and rapidly growing literature devoted to the question of adherence (Moran et al., 2021; Noone et al., 2021). Although there are multiple factors beyond psychological motivation which impact adherence (for instance, in order to abide by COVID‐19 measures, people need both the necessary information and the necessary resources to do so—see Michie et al., 2011; Reicher & Drury, 2021), and whereas there are multiple psychological determinants of adherence (van Bavel et al., 2020), one consideration which has consistently been found to be of importance is the perception of risk. This has been shown across a range of pandemics (Kim et al., 2015; Rubin et al., 2009; Tang & Wong, 2004; Webster et al., 2020). The effects of risk perception have been demonstrated during the COVID‐19 pandemic across a range of behaviors, influencing, for example, hand washing (Cabrera‐Alvarez et al., 2022; Wise et al., 2020), physical distancing (Abu‐Akel et al., 2021; Rothgerber et al., 2020; Wise et al., 2020), self‐isolation and lockdown adherence (Siegrist et al., 2021; Smith et al., 2020), vaccination intentions and uptake (Butter et al., 2022; Caserotti et al., 2021), and engagement with relevant media (Zhou et al., 2022).

At one level, this makes good sense—after all, unless something constitutes a threat and hence poses a risk, why would one devote efforts to mitigating against it, especially if one is required to act in ways that are onerous (such as self‐isolating) and restrictive (such as physical distancing). At the same time however, the existing evidence is limited in two important ways.

The first limit is conceptual. Research on (perceived) risk and adherence generally fails to address who is (perceived to be) at risk. It either presupposes that risk means risk to the individual self (as opposed to other individuals) or else it does not address the question at all. Yet, over recent years, social psychologists have made a distinction between personal identity and social identity, the former referring to defining oneself in individual terms, the latter to defining oneself in terms of membership of a social group (Reicher et al., 2010; Tajfel, 1974). In addition to personal identity, we all have a range of collective (or social) identities corresponding to the different groups we belong to which will be salient in different contexts. Moreover, which identity is salient is crucial to the cognitions, emotions, and behaviors of the actor. Notably, when social identities are salient, people will act in terms of the norms, values, and interests of their group and not of themselves as individuals (Turner et al., 1987).

In the context of risk, this would imply that our estimates of the threat posed by any specific phenomenon could vary considerably as a function of whether we think in terms of personal or social identity. Something that is of little risk to us as individuals may be of great risk to the wider group and vice versa. Therefore, in estimating the impact of risk upon behavior we must ask “which risk?”—or rather, to be more precise, “risk in relation to which identity?”.

The overall relevance of social identification processes in the COVID‐19 pandemic has been amply demonstrated, notably by a range of studies showing that social identification at various levels (local community, state, and nation) is related to adherence to mitigation measures (Cardenas et al., 2021; Stevenson et al., 2021; van Bavel et al., 2022; Vignoles et al., 2021). In this article, we are specifically interested in the relevance of social identification processes to the perception of COVID risk. There is already evidence that the pandemic is viewed as riskier to the community than to oneself (Siegrist et al., 2021) but do people think in terms of the community risk rather than their personal risk when making decisions about physical distancing, getting tested, and other preventative behaviors? To put it slightly differently, is it risk to the personal self or to the group which impacts adherence to COVID mitigations? The question is not only of theoretical interest. It also has practical implications for the design of public health messaging.

The second limit of research on risk and adherence is the widespread reliance on self‐report measures of behavior—as is true more generally in COVID‐19 research (Davies et al., 2022). These measures are subject to a series of limitations, such as social desirability biases, selective recall, and problems of judgment (Prince et al., 2008; Shephard, 2003). These are not reasons for simply rejecting self‐reports and indeed the problems are clearly greater with some behaviors than others, such as those where actors are less observed and where judgments of compliance are less clear cut. Thus, in a study of adherence during the COVID‐19 pandemic, Davies et al. (2023) showed that self‐report measures are highly discrepant from observational data for some behaviors (handwashing and physical distancing) but not for others (mask wearing in public spaces).

Although Davies et al. (2023) made an understandable call for more observational research, the obvious problem is that this is not always practical or indeed possible, especially where the behaviors themselves are private or else (as in the present case) there is a need to collect additional non‐observable data. One alternative is to exploit the possibilities of digital technologies. A number of studies have demonstrated the effectiveness and validity of immersive Virtual Reality techniques in studying social interaction (e.g., Gonzales‐Franco et al., 2018; Pan & Hamilton, 2018), including for phenomena which are difficult to research (e.g., Gonzalez‐Franco et al., 2018; Rovira et al., 2021). Other studies have developed simpler and more accessible computer‐based versions of the behaviors that concern them. Luckman et al. (2021), for example, used a computer‐based task in their work on the effects of face mask usage on physical distancing in a COVID‐19 context. In different stylized scenarios, participants were required to select the minimum (horizontal) distance they would position a figure representing themselves from a figure representing a stranger. The distance was lower if either figure was wearing a mask. We follow a similar approach here, developing novel, intuitive, and computer‐based tasks to study our behaviors of interest.

We report two studies which address the impact of (a) perceived individual risk, and (b) perceived collective risk on two key behaviors necessary to the reduction of COVID‐19 transmission. The first study looks at physical distancing (how far participants keep from others). The second study looks at testing for COVID‐19 (how quickly people participants choose to get a test after symptoms start developing).

## 2 STUDY 1

For this first study, we focussed on the effect of individual and collective risk perceptions on physical distancing, one of the components of the “Hands. Face. Space” campaign in the United Kingdom.

To measure distancing, we have developed an intuitive, interactive task in which participants position an avatar of themselves in relation to others in different scenarios. At the time of data collection, 27 May 2021, the UK guidelines were to be mindful of surroundings and keep a 2 m distance from people who were not members of your household.

### 2.1 Methodology

We first collected 6 single‐item measures of participants’ perceptions of COVID‐19 risk: the risk to themselves, other people in their household, their family and close friends, and their local, national, and world communities. We also collected their vaccination status and their sense of shared identity with their local community. See Section S1.1.1 for details.

The participants then took part in a physical distancing task. They were presented with 20 trials in which they indicated where they would position themselves relative to another individual in different hypothetical scenarios. This allowed us to investigate how a participant's physical distancing was affected by (their perceptions of) different types of risk. We also manipulated whether the other individual was a friend or a stranger (anticipating less physical distancing between friends in‐line with the “Friend‐Shield” account; De Vries & Lee, 2022), and whether they were or were not wearing a mask (anticipating less physical distancing when the other individual was wearing a mask in‐line with a “risk compensation” account; Luckman et al., 2021). The first 16 trials involved scenarios in which both the participant and the other individual were standing. In the final 4 trials, they were both seated. Our trials bear similarity to those of Luckman et al. (2021), discussed above, although we used a different set of scenarios, and our distances were based on both the horizontal and vertical positions of the figures on the screen, allowing more variability in where the participant could place their figure. The task was developed using *jsPsych* (de Leeuw, 2015).

#### 2.1.1 Standing trials

In each of the 16 standing trials, the participant was presented with a bounded area (550 × 550 pixels in size). A cartoon figure of a person (150 pixels in height), representing another individual, was in the center of this area, and could not be moved by the participant. A second figure—another cartoon of a person (also 150 pixels in height), wearing a blue t‐shirt with a “YOU” logo on it—represented the participant. Using their mouse to click and drag their figure, the participant was required to position it to indicate where they would stand relative to the other individual in each scenario. We then measured the distance in pixels from the center of the participant figure's face to the center of the other figure's face. The maximum possible distance was 495 pixels. For reference, if we interpret the figures’ heights of 150 pixels as 169 cm (the mean of the UK average male [175 cm] and female [162 cm] heights), then 178 pixels would be equivalent to a 2 m physical distance.

**FIGURE 1:**
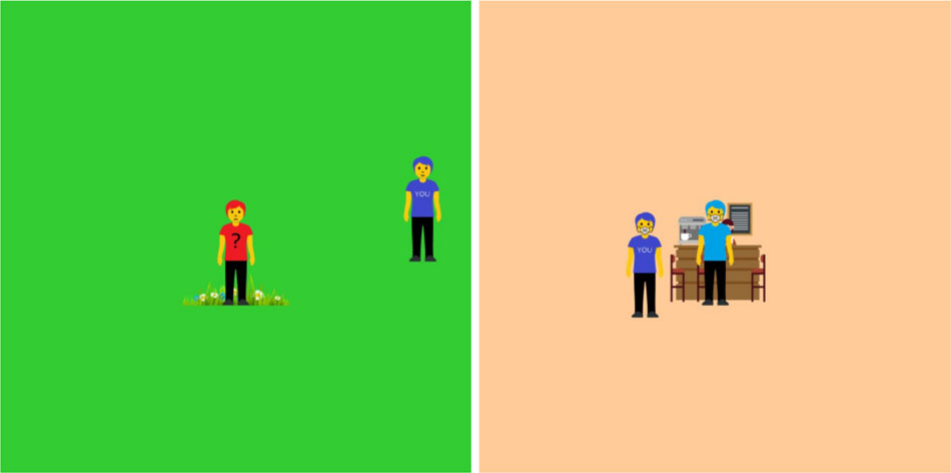
Example standing physical distance trials. The left image shows the participant's figure talking to a stranger who is not wearing a mask in a park. The distance between them is 267 pixels. The right image shows the participant's figure queuing behind a friend who is wearing a mask in a cafe. The distance between them is 99 pixels.

Each of the 16 standing trials involved a different scenario, unique in its combination of (i) which of 4 different locations the figures were presented in, (ii) whether or not the other individual was wearing a mask, and (iii) whether or not the other individual was a friend or a stranger. The four different locations, which were used to introduce some contextual variability including a mix of public/private and indoor/outdoor settings, were: a park, where the participant's figure was talking to the other individual; a cafe, where the participant's figure was queuing behind the other individual; a room in the participant's home, where the participant's figure was talking to the other individual about repairs; a friend's garden, where the participant's figure was talking to the other individual. In the indoor scenarios (the cafe and in the room in their home), the participant's figure was wearing a mask. In the outdoor scenarios, they were not wearing a mask. For each of the 4 trials involving a given location, the participant's figure was the same, but we used one of 4 different figures for the other individual. One trial had the other figure being a friend wearing a mask, one a friend not wearing a mask, one a stranger wearing a mask, and one a stranger not wearing a mask. See Figure 1 for screenshots illustrating two different scenarios.

The trials were presented in a random order for each participant, and the participant's figure's starting position was randomly determined for each trial. When the participant was happy with the position of their figure, they advanced to the next trial using a continue button. If the participant's figure was not touching the bounded area, they were prompted to move their figure closer to the other individual and were unable to progress until they had done so. Before the first scenario, the participant was given some practice in clicking and dragging their figure to ensure they understood what was required of them.

#### 2.1.2 Seated trials

**FIGURE 2:**
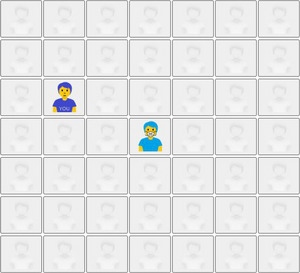
Example seated physical distance trial. In this scenario, the participant was required to select a seat in a doctor's waiting room where the other individual was a friend wearing a mask. They have chosen a seat which places them at a distance of 2.24 seats.

For each of the 4 seated trials, the participant was presented with a 7 × 7 grid of tiles representing seats in a doctor's waiting area. The center seat was taken by another individual. The participant's task was to select the seat they would sit in. One trial had the other individual as a friend wearing a mask, one as a friend not wearing a mask, one a stranger wearing a mask, and one a stranger not wearing a mask. The trials were presented in a random order for each participant. An example trial is illustrated in Figure 2.

Seated physical distance was taken as the distance between the center points of each seat, using seat as unit of measurement (e.g., if the participant had left 1 empty seat between them and the other individual but sat in the same row, the distance would be 2 seats, although if they had sat in one of the closest seats directly diagonal to the other individual, the distance would be *√*2 = 1.414 seats; the maximum distance was 4.243 seats).

Note that throughout both the standing and seated physical distancing trials, all the figures had the same haircut and the same yellow skin color. We did not intend the figures to represent people of a particular gender or skin color (and so, rather than privileging one skin color over others, we selected yellow skin following its typical use in the emojis of human figures to indicate individuals who could be of any skin color). It is, however, possible that our participants varied in how much they perceived the figures as comparatively in‐group or out‐group, and that this may have influenced their perceptions of risk in a way which affected their avatar placements (see Yee & Bailenson, 2007, for relevant discussion of the Proteus Effect). We do not anticipate that this would have affected our overall results, however, as figure appearance was held constant across our (within‐participant) manipulations, and we controlled for gender in our statistical analyses.

After the physical distancing tasks, we used response scales to collect additional, reported, data on the participants’ intentions to take other preventative measures in the next week and the measures they had taken in the previous week. See Section S1.1.2 for details. We also collected the participant's age, gender, and pretax income (in £10,000 bands) to control for the effects these variables in our analyses, previous work having indicated associations between them and adherence with preventative behaviors (e.g., Smith et al., 2021, with respect to adherence to the test, trace, and isolate system in the United Kingdom). We collected participant location as a check that we had successfully prescreened our participants to only recruit those in the United Kingdom. As part of a wider project, we also collected additional data—employment situation, number of adults and children in their household, and the amount of time they had been working at home due to the pandemic—which is not relevant to this particular article. Participants were free to withhold any of this information.

This was an exploratory study. We wanted to see if there was a distinction between different types of risk, such as perceptions of risk to the individual and perceptions of risk to the group, and, if there were any differences, to investigate whether each predicted behavior. Though we anticipated, based on previous work (e.g., Siegrist et al., 2021), that risk would influence behavior, we did not make any firm predictions about the effects of different types of risk.

#### 2.1.3 Participants

Data from 200 participants was collected using Prolific on 27 May 2021. Each was paid £2. Though we used Prolific's prescreening to only recruit participants who had indicated that their current location was the United Kingdom, one participant reported that they were currently living in Poland, and so we removed their data from the analysis. Our final sample was therefore of 199 UK‐based participants (142 female, 54 male, and 3 other/undisclosed gender; age 18–89, mean 33.6; age distribution illustrated in Section S3.

175 participants lived in England, 13 in Scotland, 8 in Wales, and 2 in Northern Ireland, with 1 undisclosed. A total of 105 (53%) participants had had at least 1 dose of a COVID‐19 vaccine and 93 (47%) had not, with 1 undisclosed (i.e., the participant responded “don't know/prefer not to say”). A technical issue during the data collection resulted in all of one participant's physical distancing and test‐booking measures not being recorded. We retained the rest of the participant's data, however, and included it in appropriate analyses.

### 2.2 Results

Mean standing physical distance (mean of individual participant means) was 167 pixels (sd = 62.0), which would be equivalent to 1.9 m. Mean seated physical distance (mean of individual participant means) was 3.04 seats (sd = 0.667).

To investigate the effects of different types of COVID‐19 risk perception on physical distancing, we first factor analyzed the 6 risk items (see Section S1.1.1). We determined a two‐factor split through parallel analysis (using the *psych* package; Revelle, 2021). Our factor analysis (using the *lavaan* package; Rosseel, 2012) then indicated an (inter)personal risk factor of the first three items (personal risk, household risk, and family and close friends risk) and a collective risk factor of the remaining three items (local community risk, national community risk, and world risk).

For illustration purposes, mean (of individual participant means) standing and seated physical distance by low and high (inter)personal and community risk are shown in Tables 1 and 2, respectively.

**TABLE 1:** Standing physical distance means (of individual participant means) by (perceptions of) (inter)personal and collective risk, measured by distance in pixels

			Collective risk	
			Low	High
			152 (*n* = 84)	178 (*n* = 114)
(Inter)personal risk	Low	162 (*n* = 93)	148 (*n* = 54)	181 (*n* = 39)
	High	172 (*n* = 105)	158 (*n* = 30)	177 (*n* = 75)

^Note:^ (Inter)personal and collective risk are split into low (less than mean (inter)personal/collective risk) and high (greater than mean (inter)personal/collective risk) risk for illustration purposes here. Our statistical analyses, however, treated (inter)personal and collective risk as continuous variables.

**TABLE 2:** Seated physical distance means (of individual participant means) by (perceptions of) (inter)personal and collective risk, measured by seat distance

			Collective risk	
			Low	High
			2.85 (*n* = 84)	3.18 (*n* = 114)
(Inter)personal risk	Low	2.99 (*n* = 93)	2.83 (*n* = 54)	3.23 (*n* = 39)
	High	3.08 (*n* = 105)	2.89 (*n* = 30)	3.16 (*n* = 75)

*Note*: (Inter)personal and collective risk are split into low (less than mean (inter)personal/collective risk) and high (greater than mean (inter)personal/collective risk) risk for illustration purposes here. Our statistical analyses, however, treated (inter)personal and collective risk as continuous variables.

We carried out our analyses using linear mixed effects modeling. As expected, both standing and seated physical distance decreased if the other individual was a friend (for standing trials, *b *= −18.531, *SE *= 0.861, *t *= −21.523, and *p *< 0.001; for seated trials, *b *= −0.564, *SE *= 0.023, *t *= −25.043, and *p *< 0.001), or if they were wearing a mask (for standing trials, *b *= −17.117, *SE *= 0.863, *t *= −19.847, and *p *< 0.001; for seated trials, *b *= −0.181, *SE *= 0.023, *t *= −8.045, and *p *< 0.001). Higher collective risk was associated with greater standing (*b *= 12.975, *SE *= 3.650, *t *= 3.555, and *p *< 0.001) and seated (*b *= 0.127, *SE *= 0.038, *t *= 3.321, and *p *= 0.001) physical distancing, and this was the case both when the other individual was a friend and stranger and both when they were and were not wearing a mask. There was no evidence of an effect of (inter)personal risk on physical distancing. See Section S1.2 for full details.

Greater collective risk, but not (inter)personal risk, was also associated with greater adoption of the additional COVID‐19 preventative measures: The measures the participants intended to take over the next week and took over the previous week. See Section S1.2.3 for details.

Note that though we have focused on the distinction between (inter)personal and collective risk in our analyses here, an alternative approach would be to assess the specific effects of personal, as opposed to (inter)personal, risk (i.e., the participant's response to the single‐item measure “I am at risk from COVID‐19”) alongside some measure of collective risk. If we repeat our analyses, replacing (inter)personal risk with personal risk alone and taking any of local community risk, national community risk, world risk, or our factor‐analysis‐determined measure of collective risk as a measure of collective risk, we get the same pattern of results. Participants take more preventative measures, including increasing physical distancing, with higher perceptions of collective risk, but there is no evidence of any effects of personal risk.

### 2.3 Discussion

Three points emerge from the findings of this study. First, they confirm the validity of a distinction between different types of risk, organized around who is at risk. They also help us to elaborate this distinction. That is, risk to the individual self forms a factor with risk to those with whom one has relations as an individual—one's friends and family. This is separate from risk to members of the various groups to which we belong. Accordingly, we term these respectively (inter)personal and collective risk.

Second, these different types of risk relate differently to behavior. Third, and more specifically, it is collective risk rather than (inter)personal risk which impacts on physical distancing. This is true whether we are standing or seated, in the presence of a friend or a stranger, someone who is masked or unmasked. It is true whether we compare personal risk with collective risk as a whole or to risk to any specific collectivity we belong to. It is true whether we compare collective risk to (inter)personal risk as a whole (including those with whom we have individual relations) or just to our individual selves.

However, one might still ask whether these findings are specific to physical distancing or whether they apply more generally to behavioral COVID‐19 mitigations. Accordingly, in the next study we address the impact of (inter)personal and collective risk on a very different mitigation behavior.

## 3 STUDY 2

The test, trace, and isolation system can play a critical role in minimizing the spread of COVID‐19. To be most effective, it is important that individuals who are symptomatic, or have been in contact with someone infected, are tested as early as possible. In the event of a positive test result, it is also crucial that they self‐isolate. At the time of our data collection, they were also required to provide contact‐tracing information.

We primarily focus on the first step of that process here, using an interactive task to assess how long participants waited while symptoms, possibly indicative of COVID‐19, developed before they booked a test. Following our results of Study 1, we predicted that test‐booking speed would be primarily motivated by perceptions of collective risk over perceptions of (inter)personal risk.

At the time of data collection, 20 September 2021, the UK public were instructed to self‐isolate if they received a positive test result, or to self‐isolate and book a test if they either developed COVID‐19 symptoms or were a close contact of someone with COVID‐19.

### 3.1 Methodology

As in Study 1, we first collected 6 single‐item measures of the participants’ perceptions of different types of COVID‐19 risk and their vaccination status. We also collected two additional measures of whether the participants perceived COVID‐19 infection as unimportant, one measure relating to personal infection (including, e.g., the item “It doesn't matter if I catch COVID‐19”), and one measure relating to infection of the general public (including, e.g., the item “It doesn't matter if people catch COVID‐19”). See Section S2.1.1 for details.

To focus the participant on the scenario of self‐isolation, we asked them to imagine how they would feel if they tested positive for COVID‐19 and were required to self‐isolate. They briefly described (a) “the financial challenges (e.g., due to reduced income),” (b) “the practical challenges (e.g., related to getting food, essentials, and medicines, or caring responsibilities),” and (c) “any other challenges (e.g., emotional)” they would face if self‐isolating.

They were then presented with details of the current (at time of data collection) £500 support package which could be available to them if they tested positive for COVID‐19 and were required to self‐isolate. They were informed that they may be eligible for this support if they were employed or self‐employed, they could not work from home, and they received benefits or were on a low income (as defined by their local council). We then asked how likely they thought they were to be eligible for this support (7‐point response scale from “Extremely unlikely” to “Extremely likely”).

**FIGURE 3:**
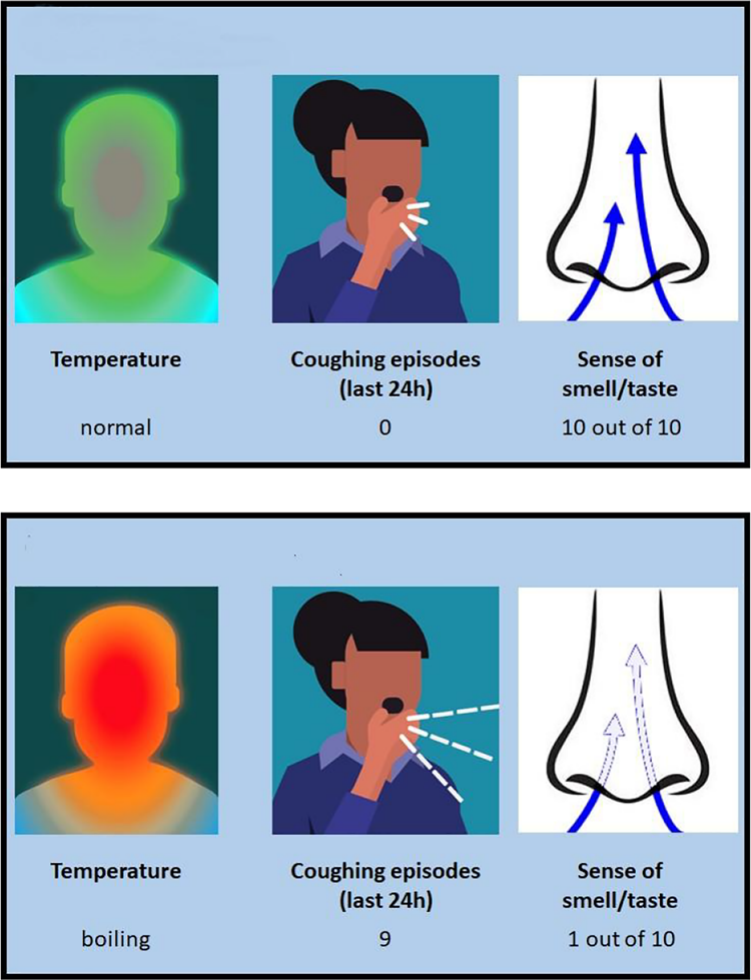
Introduction to COVID‐19 symptoms. Before the test‐booking trials, the participants were told the main three symptoms of COVID‐19 as used by the NHS. The image in the top panel was used to illustrate a person with no symptoms of COVID‐19. The image in the bottom panel was used to illustrate a person with severe symptoms of COVID‐19.

They were then asked the extent to which they agreed that it would be difficult to self‐isolate due to financial, practical, and other (e.g., emotional) challenges, each on a 7‐point scale. The participant then completed the interactive test‐booking task (which was again developed using *jsPsych*). They were first given a summary of the symptoms of COVID‐19 as used by the NHS (at the time of data collection), that is, that there are three main symptoms—a high temperature, a new, continuous cough, and a loss or change to your sense of smell or taste—and that a test should be booked if any of them are present. We specified that the test referred to was a PCR test which would be sent away to a lab, as opposed to a test that could be completed at home. The participants were then shown a three‐panel visual representation of a person having none of the symptoms to any extent (a temperature described as “normal,” 0 coughing episodes over the last 24 h, and a sense of smell/taste described as “10 out of 10”; see Figure 3, top panel), followed by an equivalent three‐panel visual representation of a person having all of the symptoms to a severe extent (a temperature described as “boiling,” 9 coughing episodes over the last 24 h, and a sense of smell/taste described as “1 out of 10”; see Figure 3, bottom panel).

**FIGURE 4:**
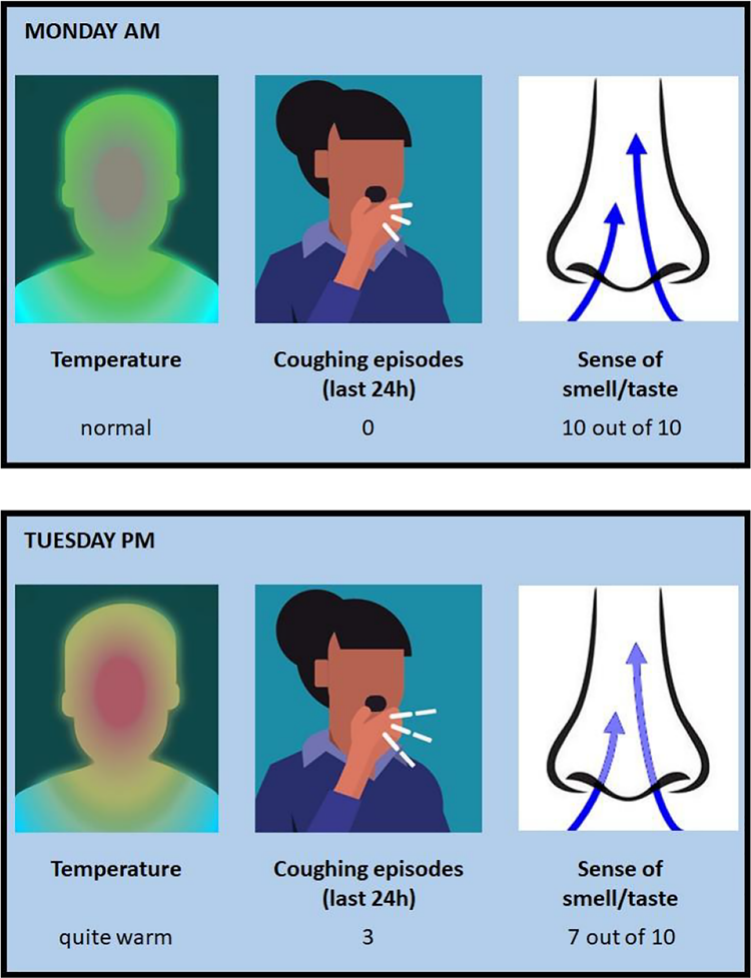
Test‐booking trial example. The top image is the visualization given to the participant at the start (Step 0) of the trial involving all three symptoms. Once the participant started the trial, this visualization remained on the screen for 3 s, before changing to the Step 1 visualization (not shown). If the participant did not elect to book a test, the symptoms continued to worsen every 3 s. The bottom image is the visualization at Step 4.

The participants then undertook 4 trials. In the first trial only temperature symptoms were present, in the second only coughing symptoms, and in the third only loss of smell/taste symptoms. In the fourth trial, all three symptoms were present together. For each trial the participant was told that they had no symptoms at the start of the week (Step 0, displayed as “Mon am” [indicating Monday morning]). When they were ready to begin the trial, the symptoms worsened every 3 s. At any point the participant could either click a button to “book a test” or wait to see how the symptoms developed. The trial ended either when the participant elected to book a test, or if they did not book a test before the end of the final step (Step 9, displayed as “Fri pm”). See Figure 4 for an illustration of the trial involving all four symptoms at Steps 0 and 4.

Our measurement of test booking was the step number at which the participant booked a test. This was treated as an ordinal variable, with Step 10 recorded for trials in which no test was booked. After the test‐booking task, we used response scales to collect additional, reported, data on the participants’ intentions to provide contact‐tracing details and self‐isolate in the event of a positive COVID‐19 test. See Section S2.1.2 for details.

As for Study 1, we also collected the participant's age, gender, and pretax income (in £10,000 bands) to control for the effects these variables in our analyses. We collected participant location as a check that we had successfully prescreened our participants to only recruit those in the United Kingdom. As part of a wider project, we also collected additional data—the participant's employment situation, number of adults and children in their household, caring responsibilities, and the amount of time they had been working at home during the pandemic—which is again not relevant to this particular article. Participants were free to withhold any of this information.

Optional text boxes were available at regular intervals for participants to comment on any of their responses if they wished, and there was additional space at the end of the study for them to add anything else they wanted to tell us about their experience of COVID‐19 or to provide feedback on the study. Two attention checks were included (one within the COVID‐19 risk items and one within the contact‐tracing items, where the participant was instructed to select “Strongly agree” and “Strongly disagree,” respectively).

Based on the results of Study 1, we predicted that there would again be a distinction between (inter)personal risk and collective risk, and that while collective risk would predict the adoption of the preventative behavior, (inter)personal risk would not.

#### 3.1.1 Participants

We collected data from 601 UK‐resident participants via Prolific on 20 September 2021. Prolific's prescreening was used to prevent participants from Study 1 from taking part. To get a spread of participants of different ages, we also prescreened to collect data from 200 participants aged 29 and under, 200 participants aged 30–59, and 200 participants aged 60 and over. We inadvertently collected data from one additional participant in the aged 30–59 category, due to their initially having trouble submitting their responses at the end of the study and another participant having been automatically recruited to take their place. Each participant was paid £2.

Data from 48 participants who failed one or both of the two attention checks was excluded. Our final sample therefore included data from 553 participants (383 female, 166 male, and 4 other/undisclosed gender; age 18–88, mean 43.1, excluding one participant who did not disclose their age; age distribution illustrated in Section S3). A total of 475 lived in England, 43 in Scotland, 27 in Wales, and 7 in Northern Ireland, with 1 undisclosed.

A total of 504 (91%) participants had had at least 1 dose of a COVID‐19 vaccine and 46 (8%) had not, with 3 (1%) undisclosed (i.e., the participant responded “don't know/prefer not to say”). A total of 131 (24%) participants had caring responsibilities for at least 1 child aged 15 and under, and 419 (76%) did not, with 3 (1%) undisclosed. A total of 56 (10%) participants had caring responsibilities for at least 1 adult, and 489 (88%) did not, with 8 (1%) undisclosed.

### 3.2 Results

Mean test‐booking step (mean of individual participant means if we treat test‐booking step as a continuous variable) was 4.91 (sd = 1.77). We followed the same approach as in Study 1 to investigate the effects of different types of (perceptions of) COVID‐19 risk on test‐booking. Parallel and factor analysis indicated the same two‐factor split: an *(inter)personal risk* factor of the first three items (personal risk, household risk, and family and close friends risk) and a *collective risk* factor of the remaining three items (local community risk, national community risk, and world risk).

For illustration purposes, mean (of individual participant means) test‐booking step by low and high (inter)personal and collective risk is shown in Table 3.

**TABLE 3:** Test‐booking step means (of individual participant means) by (perceptions of) low and high (inter)personal and collective risk

			Collective risk	
			Low	High
			5.09 (*n* = 251)	4.76 (*n* = 299)
(Inter)personal risk	Low	5.03 (*n* = 261)	5.20 (*n* = 173)	4.71 (*n* = 88)
	High	4.80 (*n* = 289)	4.86 (*n* = 78)	4.78 (*n* = 211)

*Note*: (Inter)personal and collective risk are split into low (less than mean (inter)personal/collective risk) and high (greater than mean (inter)personal/collective risk) for illustration purposes here. Our statistical analyses, however, treated test‐booking step as an ordinal variable and (inter)personal and collective risk as continuous variables.

We carried out our analyses using cumulative link mixed effects modeling. Higher perceptions of collective risk were associated with earlier test booking (*b *= −0.191, *SE *= 0.088, *z *= −2.175, and *p *= 0.030). There was no evidence of any effects of perceptions of (inter)personal risk on test‐booking step (*b *= 0.022, *SE *= 0.076, *z *= 0.286, and *p *= 0.775). See Section S2.2 for full details.

The additional data on the participants’ intentions to provide contact‐tracing details and self‐isolate in the event of a positive test indicated that increased perceptions of collective risk and, contra the other findings we present here, increased perceptions of (inter)personal risk were both associated with greater adoption of preventative measures. See Section S2.2.1 for details.

Of course, there may be other factors, such as employment type, which prevent higher risk individuals from being able to comply with authority guidelines (e.g., Sheth & Wright, 2020). Exploratory analyses of participant personal circumstances also suggested that lower household income and older age were associated with waiting longer until booking a test. See Section S2.2.2 for details, where we also include additional details about the role of personal circumstances on the difficulties of self‐isolation.

As in Study 1, we have focussed on the distinction between (inter)personal and collective risk in our analyses here, but an alternative approach would be to assess the specific effects of personal risk (i.e., the participant's response to the single‐item measure “I am at risk from COVID‐19”) alongside collective risk. If we repeat the analyses above, replacing (inter)personal risk with personal risk alone and taking any of local community risk, national community risk, world risk, or our factor‐analysis‐determined measure of collective risk as a measure of collective risk, we get the same pattern of results. Participants take more preventative measures, including booking a test sooner, with higher perceptions of collective risk, but there is no evidence of any effects of personal risk.

We also get the same pattern of results for test booking if, instead of investigating the effects of different risk perceptions, we use measures of how much it matters if the participant gets infected and how much it matters if people in general get infected (see Section S2.1.1 for items). Consistent with our findings for (inter)personal risk and collective risk, seeing general infection as important reduced test‐booking steps, whereas there was no evidence that seeing personal infection as important had any effect. See Section S2.2.3 for details.

### 3.3 Discussion

The findings of this second study very largely corroborate those of the first. Once again we get a distinction between risk to the individual/those with whom one has individual relations ((inter)personal risk) and risk to members of the various communities with which one is associated (collective risk). Again, (inter)personal risk and collective risk have different relations to behavior. And again, it is, by and large, collective rather than (inter)personal risk which is associated with greater adherence (getting a COVID‐19 test sooner after the onset of symptoms).

The one exception to this pattern is that both (inter)personal and collective risk relate to the expressed intention to adhere to other elements of the test, trace, and isolate process (providing contact‐tracing details and self‐isolate in the case of a positive test). However, it is, perhaps, telling, that these are measured through self‐report rather than computerized behavioral simulation.

In combination, however, the two studies show that, across two very different behaviors, it is the perception of collective risk—that is risk to the groups to which one belongs—that impacts adherence. Let us finish by considering the general implications of these findings.

## 4 GENERAL DISCUSSION

This article had two aims. First to develop our understanding of the relationship between risk and adherence by addressing whether, in the context of adherence to COVID mitigations, people think in terms of personal or social identity (and hence of risk to their individual self or to members of the group they identify with). The answer from both studies is clear. Whether it is a matter of physical distancing or of taking a COVID test as symptoms develop, our respondents adhered as a function of the level of risk to the group and not because they or their friends or their family were perceived to be at risk. It is worth noting that this common finding was observed both in the presence of other people (avatars) in Study 1, and in the absence of other people in Study 2. This is unsurprising given that social identity processes do not depend on the presence of others since social identities may be salient even when individuals are alone and thus shaping their psychology and actions.

It is important to be clear here. Our argument concerns the importance of distinguishing between these different types of risk associated with different levels of identity (the interpersonal and the collective) and addressing which is relevant in any given context for shaping behavior. It is not to claim that collective risk will always trump (inter)personal risk. To the contrary, the social identity approach concerns the variability of the self‐concept—and hence of associated cognitions, emotions, and behaviors—in different contexts. Consequently, whether collective risk trumps (inter)personal risk, or vice‐versa, will depend upon which identity is salient in the context of concern.

Our specific findings, we suggest, are due to the fact that in the early stages of the pandemic (when our data was collected) the stress was predominantly on collective identity and the need to act in ways that kept everyone in the community safe. Thus, in his letter of March 2020, sent to all households in the United Kingdom (and hence to all our participants), Prime Minister Boris Johnson praised those health care workers and volunteers who were helping the most vulnerable and continued: “It is with that great British spirit that we will beat coronavirus and we will beat it together” (Civil Service World, 2020). As the emphasis shifts from the collective to the individual—from social solidarity to personal responsibility—later in the pandemic, we would expect the relative impact of collective and (inter)personal risk to shift from the former to the latter.

Of course, in order to validate this suggestion, it will be necessary to conduct future studies in which we manipulate the relative salience of different identities and look at the impact on different types of risk and their relationship to adherence. For now, there are two important implications of the argument we wish to emphasize. The first is conceptual and concerns both how we think of the social identity approach and of risk. Social identity is often thought of as a social psychological theory both in terms of the explanans (how it seeks to explain phenomena) and the explanandum (what phenomena it seeks to explain). Hence, it is seen as limited to such phenomena as social stereotyping, social influence, group relations, and so on. However, this is a misunderstanding. Although the explanatory focus is on the structure of the social self in context, the theory was always directed toward an explanation of human understanding in general (Turner et al., 1994). Accordingly, Xiao et al. (2016) documented the way in which social identification impacts on the various forms of perception—visual, auditory, olfactory, tactile, and gustatory. They acknowledge that most of the work is in the visual domain but to invoke just one exception from our own work, people find the smell of sweaty t‐shirts less disgusting if they are in‐group (i.e., bearing the logo of participants own University) than out‐group (Reicher et al., 2016).

However, we can go further. The full radicalism of a social identity approach becomes clear once we realize that the notion of a variable self necessarily problematizes all self‐related concepts (Reicher & Hopkins, 2016). We can no longer ask about, say, self‐esteem or self‐efficacy or self‐realization. We always have to ask “what is the self of self‐esteem, self‐efficacy, self‐realisation?”. The implications are, perhaps, most profound when we address two concepts that are foundational in psychology and the wider human sciences: self‐relevance and self‐interest. Thus, whether an event holds any significance for ourselves, whether we respond to it, attend to it, devote any cognitive resource to it may depend on whether we think of “ourselves” individually or collectively (McClung & Reicher, 2018) and in terms of what collectivity. For Britons, an earthquake in Italy will be of less relevance and concern if they think of themselves in terms of national rather than European identity (Levine & Thompson, 2004).

Similarly, whether and how an event impacts our interests (and hence what sort of risk the event represents) will be a function of identity salience. This is not only a matter of who is affected (impact on fellow group members affecting evaluations when group membership is salient) but also how. Thus, for instance, the risk of a visually disfiguring condition is taken more seriously when gender identity is salient, whereas the risk of a physically disabling condition is taken more seriously when sporting identity is salient (Levine & Reicher, 1996).

In all these various ways, we suggest that evaluations of risk will vary as a function of identity salience. Risk is always risk to someone or something. Risk calculations involve an implicit self term. Hence, we cannot answer questions of risk without asking “what self?”. This is not just a conceptual issue. As we saw during the COVID‐19 pandemic, it has life‐and‐death practical implications. That is, if people were to focus purely on personal risks, many who are in relatively low‐risk groups (in the case of COVID‐19, younger age‐groups and those without comorbidities) may be disinclined to adhere to mitigation measures. However, if they were to consider collective risk and the ways that their actions might pose a severe risk to vulnerable members of their community, adherence would be more likely. Thus, by stressing collective over personal identity it would be possible to increase adherence in otherwise recalcitrant groups. There are important implications here for health messaging (Bonnell et al., 2020; Neville et al., 2021).

Let us turn now to our second aim, which was to develop innovative methodologies which reduce our reliance on self‐report measures of adherent behavior. For each study, we developed simple and easily used computer‐based tasks: for Study 1, instead of asking people how far they would stand from others, getting them to place an avatar of themselves amongst others; for Study 2, instead of asking participants how soon people book a test after symptom onset, getting them to choose when to book a test as symptoms develop.

We do not suggest that these remove all the problems of self‐report but they do address some, particularly memory and judgment biases. Clearly, future studies would need to compare the findings using these types of tasks to both self‐report and observational data. Nonetheless, we consider that they show considerable promise as an additional methodological option to be used alongside—and triangulated with—other approaches.

In conclusion, then, we consider that our article has largely succeeded in both its aims. It shows the importance of considering the impact of different types of risk (collective and (inter)personal) on adherence and it does so using innovative methods. More generally, it points both to the conceptual importance of addressing variable selfhood in risk analysis and to the practical interventions that can help deal with COVID‐19 and other pandemics.

## Supporting information

Supporting Information

## Footnotes

1 We prefer this term to the more often used ‘social distancing’ since prevention of infection transmission depends upon physical, not social distance, and indeed maintaining social proximity is important to maintaining both physical and mental health.

## References

[risa14155-bib-0001] Abu‐Akel, A. , Spitz, A. , & West, R. (2021). Who is listening? Spokesperson effect on communicating social and physical distancing measures during the COVID‐19 pandemic. Frontiers in Psychology, 11, 564434. 33510664 10.3389/fpsyg.2020.564434 PMC7837291

[risa14155-bib-0002] Bonell, C. , Michie, S. , Reicher, S. D. , West, R. , Bear, L. , Yardley, L. , Curtis, V. , Amlôt, R. , & Rubin, G. J. (2020). Harnessing behavioural science in public health campaigns to maintain ‘social distancing’ in response to the COVID‐19 pandemic: Key principles. Journal of Epidemiology and Community Health, 74(8), 617–619. 32385125 10.1136/jech-2020-214290 PMC7368244

[risa14155-bib-0003] Butter, S. , McGlinchey, E. , Berry, E. , & Armour, C. (2022). Psychological, social, and situational factors associated with COVID‐19 vaccination intentions: A study of UK key workers and non‐key workers. British Journal of Health Psychology, 27(1), 13–29. 33949038 10.1111/bjhp.12530 PMC8236922

[risa14155-bib-0004] Cabrera‐Álvarez, P. , Hornsey, M. J. , & Lobera, J. (2022). Determinants of self‐reported adherence to COVID‐19 regulations in Spain: Social norms, trust and risk perception. Health Promotion International, 38, 1–9. 10.1093/heapro/daac138 PMC9620366 36300700

[risa14155-bib-0005] Cárdenas, D. , Orazani, N. , Stevens, M. , Cruwys, T. , Platow, M. , Zekulin, M. , & Reynolds, K. J. (2021). United we stand, divided we fall: Sociopolitical predictors of physical distancing and hand hygiene during the COVID‐19 pandemic. Political psychology, 42(5), 845–861. 34548715 10.1111/pops.12772 PMC8447036

[risa14155-bib-0006] Caserotti, M. , Girardi, P. , Rubaltelli, E. , Tasso, A. , Lotto, L. , & Gavaruzzi, T. (2021). Associations of COVID‐19 risk perception with vaccine hesitancy over time for Italian residents. Social Science & Medicine, 272, 113688. 33485215 10.1016/j.socscimed.2021.113688 PMC7788320

[risa14155-bib-0007] Civil Service World . (2020). Read in Full: Boris Johnson's Letter to the Nation on Coronavirus. Accessed from: https://www.civilserviceworld.com/professions/article/read‐in‐full‐boris‐johnsons‐letter‐to‐the‐nation‐on‐coronavirus

[risa14155-bib-0008] Davies, R. , Mowbray, F. , Martin, A. F. , Smith, L. E. , & Rubin, G. J. (2022). A systematic review of observational methods used to quantify personal protective behaviours among members of the public during the COVID‐19 pandemic, and the concordance between observational and self‐report measures in infectious disease health protection. BMC Public Health, 22(1), 1–13. 35902818 10.1186/s12889-022-13819-0 PMC9330943

[risa14155-bib-0009] Davies, R. , Weinman, J. , & Rubin, G. J. (2023). Observed and self‐reported COVID‐19 health protection behaviours on a university campus and the impact of a single simple intervention. medRxiv. https://pubmed.ncbi.nlm.nih.gov/36694345/ 10.1093/pubmed/fdac147 PMC10470332 36694345

[risa14155-bib-0010] de Leeuw, J. R. (2015). jsPsych: A JavaScript library for creating behavioral experiments in a web browser. Behavior Research Methods, 47(1), 1–12. 24683129 10.3758/s13428-014-0458-y

[risa14155-bib-0011] De Vries, E. L. E. , & Lee, H. C. (2022). Friend‐shield protection from the crowd: How friendship makes people feel invulnerable to COVID‐19. Journal of Experimental Psychology: Applied, 28, 794–815. 35389684 10.1037/xap0000417

[risa14155-bib-0012] ECDC . (2021). Non‐Pharmaceutical Interventions against COVID‐19. Retrieved from https://www.ecdc.europa.eu/en/covid‐19/prevention‐and‐control/non‐pharmaceutical‐interventions

[risa14155-bib-0013] Gonzalez‐Franco, M. , Slater, M. , Birney, M. E. , Swapp, D. , Haslam, S. A. , & Reicher, S. D. (2018). Participant concerns for the learner in a virtual reality replication of the Milgram obedience study. PLoS One, 13(12), e0209704. 30596731 10.1371/journal.pone.0209704 PMC6312327

[risa14155-bib-0014] Kim, Y. , Zhong, W. , Jehn, M. , & Walsh, L. (2015). Public risk perceptions and preventive behaviors during the 2009 H1N1 influenza pandemic. Disaster Medicine and Public Health Preparedness, 9(2), 145–154. 25882121 10.1017/dmp.2014.87

[risa14155-bib-0015] Levine, R. M. , & Reicher, S. D. (1996). Making sense of symptoms: Self‐categorization and the meaning of illness and injury. British Journal of Social Psychology, 35, 245–256. 8689097 10.1111/j.2044-8309.1996.tb01095.x

[risa14155-bib-0016] Levine, M. , & Thompson, K. (2004). Identity, place, and bystander intervention: Social categories and helping after natural disasters. The Journal of Social Psychology, 144, 229–245. 15168427 10.3200/SOCP.144.3.229-245

[risa14155-bib-0017] Luckman, A. , Zeitoun, H. , Isoni, A. , Loomes, G. , Vlaev, I. , Powdthavee, N. , & Read, D. (2021). Risk compensation during COVID‐19: The impact of face mask usage on social distancing. Journal of Experimental Psychology: Applied, 27(4), 722–738. 35073133 10.1037/xap0000382

[risa14155-bib-0018] McClung, J. S. , & Reicher, S. D. (2018). Representing other minds: Mental state reference is moderated by group membership. Journal of Experimental Social Psychology, 76, 385–392.

[risa14155-bib-0019] Michie, S. , Van Stralen, M. M. , & West, R. (2011). The behaviour change wheel: A new method for characterising and designing behaviour change interventions. Implementation Science, 6(1), 1–12. 21513547 10.1186/1748-5908-6-42 PMC3096582

[risa14155-bib-0020] Moran, C. , Campbell, D. J. , Campbell, T. S. , Roach, P. , Bourassa, L. , Collins, Z. , Stasiewicz, M. , & McLane, P. (2021). Predictors of attitudes and adherence to COVID‐19 public health guidelines in Western countries: A rapid review of the emerging literature. Journal of Public Health, 43(4), 739–753. 33704456 10.1093/pubmed/fdab070 PMC7989238

[risa14155-bib-0021] Neville, F. G. , Templeton, A. , Smith, J. R. , & Louis, W. R. (2021). Social norms, social identities and the COVID‐19 pandemic: Theory and recommendations. Social and Personality Psychology Compass, 15, e12596. 34230834 10.1111/spc3.12596 PMC8250129

[risa14155-bib-0022] Noone, C. , Warner, N. Z. , Byrne, M. , Durand, H. , Lavoie, K. L. , McGuire, B. E. , McSharry, J. , Meade, O. , Morrissey, E. , Molloy, G. J. , O'Connor, L. , & Toomey, E. (2021). A scoping review of research on the determinants of adherence to social distancing measures during the COVID‐19 pandemic. Health Psychology Review, 15(3), 350–370. 34027798 10.1080/17437199.2021.1934062

[risa14155-bib-0023] Pan, X. , & Hamilton, A. F. D. C. (2018). Why and how to use virtual reality to study human social interaction: The challenges of exploring a new research landscape. British Journal of Psychology, 109(3), 395–417. 29504117 10.1111/bjop.12290 PMC6055846

[risa14155-bib-0024] Prince, S. A. , Adamo, K. B. , Hamel, M. E. , Hardt, J. , Gorber, S. C. , & Tremblay, M. (2008). A comparison of direct versus self‐report measures for assessing physical activity in adults: A systematic review. International Journal of Behavioral Nutrition and Physical Activity, 5(1), 1–24. 18990237 10.1186/1479-5868-5-56 PMC2588639

[risa14155-bib-0025] Revelle, W. (2021). Psych: Procedures for Psychological, Psychometric, and Personality Research. http://CRAN.R‐project.org/package=psych

[risa14155-bib-0026] Reicher, S. , & Hopkins, N. (2016). Perception, action, and the social dynamics of the variable self. Psychological Inquiry, 27, 341–347.

[risa14155-bib-0027] Reicher, S. D. , Templeton, A. , Neville, F. , Ferrari, L. , & Drury, J. (2016). Core disgust is attenuated by ingroup relations. Proceedings of the National Academy of Sciences of the United States of America, 113, 2631–2635. 26903640 10.1073/pnas.1517027113 PMC4790993

[risa14155-bib-0028] Reicher, S. D. , & Drury, J. (2021). Pandemic fatigue? How adherence to COVID‐19 regulations has been misrepresented and why it matters. British Medical Journal, 372, n137. 33461963 10.1136/bmj.n137

[risa14155-bib-0029] Reicher, S. D. , Spears, R. , & Haslam, S. A. (2010). The social identity approach in social psychology. In M. Wetherell , & C. Mohanty (Eds.), The sage handbook of identities (pp. 45–62). Sage.

[risa14155-bib-0030] Rovira, A. , Southern, R. , Swapp, D. , Campbell, C. , Zhang, J. J. , Levine, M. , & Slater, M. (2021). Bystander affiliation influences intervention behavior: A virtual reality study. SAGE Open, 11(3), 21582440211040076.

[risa14155-bib-0031] Rosseel, Y. (2012). lavaan: An R package for structural equation modeling. Journal of Statistical Software, 48(2), 1–36.

[risa14155-bib-0032] Rothgerber, H. , Wilson, T. , Whaley, D. , Rosenfeld, D. L. , Humphrey, M. , Moore, A. , & Bihl, A. (2020). Politicizing the COVID‐19 pandemic: Ideological differences in adherence to social distancing. PsyArXiv. https://psyarxiv.com/k23cv/

[risa14155-bib-0033] Rubin, G. J. , Amlôt, R. , Page, L. , & Wessely, S. (2009). Public perceptions, anxiety, and behaviour change in relation to the swine flu outbreak: Cross sectional telephone survey. BMJ, 339, b2651. 19574308 10.1136/bmj.b2651 PMC2714687

[risa14155-bib-0034] Shephard, R. J. (2003). Limits to the measurement of habitual physical activity by questionnaires. British Journal of Sports Medicine, 37(3), 197–206. 12782543 10.1136/bjsm.37.3.197 PMC1724653

[risa14155-bib-0035] Sheth, K. , & Wright, G. C. (2020). The usual suspects: Does risk tolerance, altruism, and health predict the response to COVID‐19? In CESifo Working Paper, 8276. 10.1007/s11150-020-09515-w PMC7585485 33132793

[risa14155-bib-0036] Siegrist, M. , Luchsinger, L. , & Bearth, A. (2021). The impact of trust and risk perception on the acceptance of measures to reduce COVID‐19 cases. Risk Analysis, 41(5), 787–800. 33438218 10.1111/risa.13675 PMC8014821

[risa14155-bib-0037] Smith, L. E. , Amlôt, R. , Lambert, H. , Oliver, I. , Robin, C. , Yardley, L. , & Rubin, G. J. (2020). Factors associated with adherence to self‐isolation and lockdown measures in the UK: A cross‐sectional survey. Public Health, 187, 41–52. 32898760 10.1016/j.puhe.2020.07.024 PMC7474581

[risa14155-bib-0038] Smith, L. E. , Potts, H. W. W. , Amlôt, R. , Fear, N. T. , Michie, S. , & Rubin, G. J. (2021). Adherence to the test, trace, and isolate system in the UK: Results from 37 nationally representative surveys. BMJ, 372, n608. 33789843 10.1136/bmj.n608 PMC8010268

[risa14155-bib-0039] Stevenson, C. , Wakefield, J. R. , Felsner, I. , Drury, J. , & Costa, S. (2021). Collectively coping with coronavirus: Local community identification predicts giving support and lockdown adherence during the COVID‐19 pandemic. British Journal of Social Psychology, 60(4), 1403–1418. 33969899 10.1111/bjso.12457 PMC8236966

[risa14155-bib-0040] Tajfel, H. (1974). Social identity and intergroup behaviour. Social Science Information, 13(2), 65–93.

[risa14155-bib-0041] Turner, J. C. , Hogg, M. , Oakes, P. , Reicher, S. D. , & Wetherell, M. (1987). Rediscovering the social group. Blackwell.

[risa14155-bib-0042] Tang, C. S.‐K. , & Wong, C.‐Y. (2004). Factors influencing the wearing of facemasks to prevent the severe acute respiratory syndrome among adult Chinese in Hong Kong. Preventive Medicine, 39(6), 1187–1193. 15539054 10.1016/j.ypmed.2004.04.032 PMC7133369

[risa14155-bib-0043] Turner, J. C. , Oakes, P. J. , Haslam, S. A. , & McGarty, C. (1994). Self and collective: Cognition and social context. Personality and Social Psychology Bulletin, 20, 454–463.

[risa14155-bib-1001] Bavel, J. J. V. , Baicker, K. , & Boggio, P. S. (2020). Using social and behavioural science to support COVID‐19 pandemic response. Nat Hum Behav 4, 460–471. 10.1038/s41562-020-0884-z 32355299

[risa14155-bib-0044] van Bavel, J. J. , Cichocka, A. , Capraro, V. , Sjåstad, H. , Nezlek, J. B. , Pavlović, T. , Alfano, M. , Gelfand, M. J. , Azevedo, F. , Birtel, M. D. , Cislak, A. , Lockwood, P. L. , Ross, R. M. , Abts, K. , Agadullina, E. , Aruta, J. J. B. , Besharati, S. N. , Bor, A. , Choma, B. L. , … Boggio, P. S. (2022). National identity predicts public health support during a global pandemic. Nature Communications, 13, 517. 10.1038/s41467-021-27668-9 PMC8792004 35082277

[risa14155-bib-0045] Vignoles, V. L. , Jaser, Z. , Taylor, F. , & Ntontis, E. (2021). Harnessing shared identities to mobilize resilient responses to the COVID‐19 pandemic. Political Psychology, 42(5), 817–826. 10.1111/pops.12726 33821062 PMC8013210

[risa14155-bib-0046] Webster, R. K. , Brooks, S. K. , Smith, L. E. , Woodland, L. , Wessely, S. , & Rubin, G. J. (2020). How to improve adherence with quarantine: Rapid review of the evidence. Public Health, 182, 163–169. 32334182 10.1016/j.puhe.2020.03.007 PMC7194967

[risa14155-bib-0047] Wise, T. , Zbozinek, T. D. , Michelini, G. , Hagan, C. C. , & Mobbs, D. (2020). Changes in risk perception and self‐reported protective behaviour during the first week of the COVID‐19 pandemic in the United States. Royal Society Open Science, 7, 200742. 33047037 10.1098/rsos.200742 PMC7540790

[risa14155-bib-0048] Xiao, Y. J. , Coppin, G. , & van Bavel, J. J. (2016). Perceiving the world through group‐colored glasses: A perceptual model of intergroup relations. Psychological Inquiry, 27, 255–274.

[risa14155-bib-0049] Yee, N. , & Bailenson, J. (2007). The proteus effect: The effect of transformed self‐representation on behavior. Human Communication Research, 33, 271–290.

[risa14155-bib-0050] Zhou, Y. , Myrick, J. G. , Farrell, E. L. , & Cohen, O. (2022). Perceived risk, emotions, and stress in response to COVID‐19: The interplay of media use and partisanship. Risk Analysis, 1–15. https://www.ncbi.nlm.nih.gov/pmc/articles/PMC9874794/ 36307383 10.1111/risa.14044 PMC9874794

