# Supplementary material for: Social identification and risk dynamics: How perceptions of (inter)personal and collective risk impact the adoption of COVID‐19 preventative behaviors
Source: Risk Anal. 2023 May 3;44(2):322–32. doi: 10.1111/risa.14155 (PMC10952649; doi:10.1111/risa.14155)
Supplement: Supplementary file 1 — Supporting Information [file RISA-44-322-s001.pdf]

Social identification and risk dynamics: how perceptions of (inter)personal and collective risk impact the adoption of COVID-19 preventative behaviours: supplementary information

Mark Atkinson[1,2], Fergus Neville[1], Evangelos Ntontis[3], and Stephen Reicher[2]

[1]School of Management, University of St Andrews
[2]School of Psychology & Neuroscience, University of St Andrews
[3]School of Psychology & Counselling, The Open University

Contents

1 Study 1 additional information 2
1.1 Additional methodological details 2
1.1.1 Perceptions of risk and shared identity with local community 2
1.1.2 Additional preventative measures 2
1.2 Analysis 3
1.2.1 Standing physical distancing 3
1.2.2 Seated physical distancing 4
1.2.3 Additional preventative measures 4

2 Study 2 additional information 4
2.1 Additional methodological details 4
2.1.1 Perceptions of risk and unimportance of infection 4
2.1.2 Self-isolation measures 5
2.2 Analysis 6
2.2.1 Preventative measures 6
2.2.2 Effect of personal circumstances on the difficulties of self-isolation and test-booking 7
2.2.3 Effect of seeing infection as unimportant 9

3 Participant age distribution 10

Bibliography 11

1 Study 1 additional information

1.1 Additional methodological details

1.1.1 Perceptions of risk and shared identity with local community

We first collected data on the participant's COVID-19 vaccination status (including number of doses, how long ago they had the latest dose, and, for the unvaccinated, willingness to have a vaccine). We then collected the following data on their perceptions of COVID-19 risk and shared identity with their local community. All items used a 7-point agreement scale from 1 ("Strongly disagree") to 7 ("Strongly agree").

Perceptions of COVID-19 risk (6 items):

- I am at risk from COVID-19.
- Other people in my household are at risk from COVID-19.
- My family and close friends are at risk from COVID-19.
- My local community is at risk from COVID-19.
- My national community is at risk from COVID-19.
- The world is at risk from COVID-19.

Local community shared identity (4 items, Cronbach's $\alpha = 0.94$):

- I have a feeling of unity with other residents of my local community.
- I have a sense of "we-ness" with other residents of my local community.
- Besides our differences, I share the same identity with other residents of my local community.
- I feel at one with the local community members around me.

1.1.2 Additional preventative measures

After they completed the physical distancing task, we asked participants about other preventative measures taken to reduce the spread of COVID-19, related to personal hygiene, travel, attending large gatherings, meeting up with other people, and intentions to self-isolate with symptoms. First, we asked about their intentions for the coming week. Second we asked about the preventative measures they had taken over the previous week. All items used a 7-point agreement scale from 1 ("Strongly disagree") to 7 ("Strongly agree").

Intentions to take preventative measures over the next week (9 items, $\alpha = 0.75$):

- I will regularly wash my hands for 20 seconds.
- I will touch my eyes, nose, or mouth with unwashed hands. (reversed item [R])
- I will travel for nonessential reasons. (R)
- I will wear a facemask in public.
- I will avoid places where many people will gather.
- I will meet up with another household outdoors. (R)
- I will meet up with another household indoors. (R)
- I will attend a large gathering. (R)
- I will self-isolate if I develop symptoms of COVID-19.

Preventative measures taken over the previous week (8 items, $\alpha = 0.65$):

- I regularly washed my hands for 20 seconds.

- I touched my eyes, nose, or mouth with unwashed hands. (R)
- I travelled for nonessential reasons. (R)
- I wore a facemask in public.
- I avoided places where many people gathered.
- I met up with another household outdoors. (R)
- I met up with another household indoors. (R)
- I attended a large gathering. (R)

1.2 Analysis

Our primary analyses investigated the effects of inter-personal risk (composite measure of the first three perceptions of COVID-19 risk items, above) and community risk (composite measures of the final three items) on each of standing physical distancing (SI Section 1.2.1), seated physical distancing (SI Section 1.2.2), and the additional preventative measures (SI Section 1.2.3).

1.2.1 Standing physical distancing

Our analysis involved linear mixed effects modelling using *lme4* (Bates et al., 2013) in *R* (R Core Team, 2021).

First, we constructed a model to confirm that physical distancing was lower when the other individual was a friend as opposed to a stranger, and when the other individual was wearing a mask as opposed to not. We included mask wearing (sum coded: 1 if the other individual was wearing a mask; -1 if they were not) and the identity of the other individual (1 if the other individual was a friend; -1 if they were a stranger) as fixed effects. Maximal random effects structures were considered in the first instance (in line with Barr et al., 2013). Our initial model therefore included random intercepts for participant identity, trial number, vaccine status (1 if the participant had had at least one dose; -1 if they had had no doses), local community shared identity (composite measure), age, gender, and household income (converted to a linear scale and log transformed). Random intercepts were removed as necessary in the event of singular fit or non-convergence (also following Barr et al., 2013).

As expected, and in line with previous work (De Vries and Lee, 2022; Luckman et al., 2020), physical distance decreased if the other individual was a friend (b = -18.531, SE = 0.861, t = -21.523, $p < 0.001$), or if they were wearing a mask (b = -17.117, SE = 0.863, t = -19.847, $p < 0.001$).

Our second model investigated the effects of inter-personal risk and community risk on physical distancing. Inter-personal risk and community risk (both centred) were included as fixed effects.[1] Our random effects structure was the same as for the first model in the first instance, though with additional random intercepts for mask wearing and the identity of the other individual.

Physical distance increased with community risk (b = 12.975, SE = 3.650, t = 3.555, $p < 0.001$), but not with inter-personal risk (b = -3.093, SE = 2.960, t = -1.045, p = 0.297).

As an additional check of the robustness of these results, we repeated our analyses on the subsets of our data where (a) the other individual was wearing a mask, (b) the other individual was not wearing a mask, (c) the other individual was a friend, and (d) the other individual was a stranger. In all cases, and despite the smaller sample sizes involved, we get the same pattern of results: physical distance increased with community risk ($b \geq 12.012$, $SE \leq 4.232$, $t \geq 3.632$, $p < 0.001$), but there was no effect of inter-personal risk ($|b| \leq 5.043$, $SE \geq 2.660$, $|t| \leq 1.896$, $p \geq 0.060$).

[1] These variables were centred but not standardised in the results we present here, but the pattern of results is the same here (and throughout our analyses) if standardised variables are used.

1.2.2 Seated physical distancing

We analysed the seated physical distancing trials in the same way as the standing physical distancing trials. Despite there being less data for the seated trials, we see the same pattern of results. Physical distance decreased if the other individual was a friend (b = -0.564, SE = 0.023, t = -25.043, $p < 0.001$), or if they were wearing a mask (b = -0.181, SE = 0.023, t = -8.045, $p < 0.001$). Physical distance increased with community risk (b = 0.127, SE = 0.038, t = 3.321, p = 0.001), but not with inter-personal risk (b = -0.028, SE = 0.031, t = -0.887, p = 0.376).

As an additional check of the robustness of these results, we again repeated our analyses on the subsets of our data where (a) the other individual was wearing a mask, (b) the other individual was not wearing a mask, (c) the other individual was a friend, and (d) the other individual was a stranger. In all cases, and despite the smaller sample sizes involved, we again get the same pattern of results: physical distance increased with community risk ($b \geq 0.097$, $SE \leq 0.048$, $t \geq 2.250$, $p \leq 0.026$), but there was no effect of inter-personal risk ($|b| \leq 0.046$, $SE \geq 0.034$, $|t| \leq 1.293$, $p \geq 0.198$).

1.2.3 Additional preventative measures

To analyse the additional preventative measures, we constructed cumulative link mixed models using the *ordinal* package (Christensen, 2019). The fixed effects were inter-personal risk and community risk (both centred). In the first instance, we included random intercepts for participant identity, specific item, local community shared identity (composite measure), age, gender, and household income (converted to a linear scale and log transformed).

Intentions to take preventative measures in the next week increased with community risk (b = 0.226, SE = 0.099, z = 2.273, p = 0.023), but not with inter-personal risk (b = 0.082, SE = 0.086, z = 0.947, p = 0.343). Similarly, reported measures taken in the previous week increased with community risk (b = 0.203, SE = 0.090, z = 2.255, p = 0.024), but not with inter-personal risk (b = 0.084, SE = 0.078, z = 1.077, p = 0.282).

2 Study 2 additional information

2.1 Additional methodological details

2.1.1 Perceptions of risk and unimportance of infection

We first collected data on the participant's COVID-19 vaccination status (including number of doses and, for the unvaccinated, willingness to have a vaccine). We then collected the following data on their perceptions of COVID-19 risk and and the extent to which the participant viewed community and personal infection as unimportant. All items used a 7-point agreement scale from 1 ("Strongly disagree") to 7 ("Strongly agree").

Perceptions of COVID-19 risk (6 items):

- I am at risk from COVID-19.
- Other people in my household are at risk from COVID-19.
- My family and close friends are at risk from COVID-19.
- My local community is at risk from COVID-19.
- My national community is at risk from COVID-19.
- The world is at risk from COVID-19.

Seeing infection as unimportant (3 items relating to infection of the general public and 3 items relating to personal infection):

- It doesn't matter if people catch COVID-19.
- The consequences of catching COVID-19 for people are not very serious.
- It is important for me to act in ways that help keep infection levels down. (R)
- It doesn't matter if I catch COVID-19.
- The consequences of catching COVID-19 for me are not very serious.
- It is important for me to act in ways that stop me getting infected. (R)

2.1.2 Self-isolation measures

The participant was asked to imagine a scenario in which they had just tested positive for COVID-19. We collected their intentions to assist with contact tracing, self-isolate from other households, and, if they shared their home with others, self-isolate within their household. All items here used a 7-point agreement scale from 1 ("Strongly disagree") to 7 ("Strongly agree").

Contact tracing (4 items, $\alpha = 0.81$):

- I would tell my employer, school, or nursery about my result (if applicable).
- To the best of my ability, I would contact everyone I had recently been in close contact with to tell them about my result.
- If asked by NHS Test and Trace, I would provide information about where I'd been recently.
- If asked by NHS Test and Trace, I would provide information about everyone I'd been in close contact with.

Self-isolation intentions, between households (5 items, $\alpha = 0.80$):

- I would fully self-isolate.
- I would never go to work, school, or public places.
- I would never go out to get food or medicines.
- I would never have (non-essential) visitors in my home.
- I would never go out to exercise.

Self-isolation intentions, within household (4 items, $\alpha = 0.92$):

- I would keep fully away from the other people in my home.
- I would eat every one of my meals on my own in my own room.
- I would avoid spending any time at all in the same room as the other people.
- I would clean any shared rooms (e.g. bathroom or kitchen) after I have used them every time.

Finally, we presented them with a set of vignettes designed to assess the extent to which they would transgress the self-isolation requirements. These items here used a 7-point likelihood scale from 1 ("Extremely unlikely") to 7 ("Extremely likely").

Self-isolation transgression vignettes (9 items, $\alpha = 0.88$):

1. There are very few people around outside at the moment. You know that you would feel a lot better if you could stretch your legs and get some fresh air by having a 10 minute walk outside. How unlikely or likely would you be to take a walk?

2. You have no way of getting any more food delivered to you today. You would really appreciate the chocolate and snacks you could quickly get from a nearby shop. How unlikely or likely would you be to take a quick trip to the shop?
3. A member of your family who lives nearby has been feeling really down recently. You'd really like to have a chat to them face-to-face to cheer them up. How unlikely or likely would you be to pop round and have a socially-distanced chat with them?
4. Your friend who lives nearby is also self-isolating. They suggest you meet up halfway between your homes to quickly swap some books and DVDs. How unlikely or likely would you be to agree to this?
5. It is early evening, and you hear that there is a spectacular meteor shower visible at the moment. You can't see it from your home, but you could if you walked 5 minutes down the road to get a perfect view. How unlikely or likely would you be to go out and see it?
6. A snack van has parked opposite your house and is selling ice creams and freshly-baked pastries. You would really like to buy something. How unlikely or likely would you be to go out to buy something from the van?
7. You see that an elderly neighbour has received a large delivery from a garden centre. They are struggling to stack a pile of boxes by their front door. How unlikely or likely would you be to go out and help them?
8. Your friend has been worried about how you have been coping since you started self-isolating, and they have come round to visit you. They are clearly expecting you to invite them in for a cup of tea. How unlikely or likely would you be to invite them in?
9. You have kept apart from the other people in your household until now, but now someone you live with is really upset. How unlikely or likely would you be to give them a hug?

2.2 Analysis

Our primary analyses investigated the effects of inter-personal risk and community risk on test-booking, supplying contact tracing information, and self-isolating (SI Section 2.2.1). We also carried out additional analyses to explore the effect of personal circumstances on the difficulties of self-isolation and test-booking (SI Section 2.2.2). Finally, we considered an alternative approach to investigating the effects of relatively (inter-)personal-level and community-level risk by investigating the effects of seeing personal and community infection as unimportant (SI Section 2.2.3).

2.2.1 Preventative measures

For each preventative measure, we ran a cumulative link mixed model (again using the *ordinal* package in *R*) with inter-personal risk and community risk (both centred) as fixed effects. In the first instance, our random effects structure included random intercepts for participant identity, trial (for the test booking measure) or item (for the other four measures), age, gender, and household income (log transformed). For the test-booking measure, we removed the 93 trials (4% of the total) where participants booked a test with no symptoms (i.e. at Step 0).

Higher community risk decreased the number of steps before a participant booked a test (b = -0.191, SE = 0.088, z = -2.175, p = 0.030). There was no effect of inter-personal risk (b = 0.022, SE = 0.076, z = 0.286, p = 0.775).

Model outputs for the other preventative measures — contact tracing, between-household self-isolation intentions, within-household self-isolation intentions, and self-isolation transgression — are given in SI Table 1.

SI Table 1: **CLMM output for other preventative measures.** Perceptions of inter-personal risk and community risk are centred.

	Risk	b	SE	z	p	
Contact tracing	Inter-personal	0.130	0.008	15.423	<0.001	***
	Community	0.443	0.009	48.172	<0.001	***
Between-household self-isolation	Inter-personal	0.142	0.008	18.209	<0.001	***
	Community	0.440	0.008	52.660	<0.001	***
Within-household self-isolation	Inter-personal	0.053	0.007	7.769	<0.001	***
	Community	0.205	0.008	27.224	<0.001	***
Self-isolation transgression	Inter-personal	-0.099	0.004	-22.388	<0.001	***
	Community	-0.314	0.005	-64.177	<0.001	***

In all cases, both higher community risk ($|b| \geq 0.205$, $SE \leq 0.009$, $|z| \geq 27.224$, $p < 0.001$) and higher inter-personal risk ($|b| \geq 0.053$, $SE \leq 0.007$, $|z| \geq 7.769$, $p < 0.001$) increased the extent to which the preventative measures were adopted.

2.2.2 Effect of personal circumstances on the difficulties of self-isolation and test-booking

To investigate the effects of personal circumstances on each of the financial, practical, and other (e.g. emotional) difficulties of self-isolation, we constructed cumulative link models with the fixed effects of age (centred), household income (log transformed and centred), childcare responsibilities (centred), adult caring responsibilities (centred), number of adults in the household (log transformed), and number of children in the household (log transformed). Note that due to not all participants disclosing all this information, these models only use data from 498 of the 553 participants.

Model outputs for financial, practical, and other difficulties of self-isolation are given in SI Tables 2, 3, and 4, respectively.

SI Table 2: **CLM output for effect of personal circumstances on financial difficulties of self-isolation.** Household income, number of adults and number of children are log transformed. Childcare and adult caring responsibilities are binary variables. Age, household income, childcare, and adult caring responsibilities are centred.

	b	SE	z	p	
age	-0.039	0.006	-6.568	<0.001	***
household income	-1.006	0.208	-4.830	<0.001	***
childcare	-0.072	0.163	-0.441	0.659	
adult caring	0.584	0.144	4.052	<0.001	***
num. adults	0.209	0.314	0.664	0.507	
num. children	0.643	0.289	2.224	0.026	*

Financial difficulties were higher for younger participants (b = -0.039, SE = 0.006, z = -6.568, $p < 0.001$), having a lower household income (b = -1.006, SE = 0.208, z = -4.830, $p < 0.001$), having adult caring responsibilities (b = 0.584, SE = 0.144, z = 4.052, $p < 0.001$), and being in a household with more children (b = 0.643, SE = 0.289, z = 2.224, p = 0.026; SI Table 2). Practical difficulties were higher for younger participants (b = -0.022, SE = 0.005, z = -4.461, $p < 0.001$), having a lower

SI Table 3: **CLM output for effect of personal circumstances on practical difficulties of self-isolation.** Household income, number of adults and number of children are log transformed. Childcare and adult caring responsibilities are binary variables. Age, household income, childcare, and adult caring responsibilities are centred.

	b	*SE*	*z*	*p*	
age	-0.022	0.005	-4.461	<0.001	***
household income	-0.460	0.188	-2.455	0.014	*
childcare	0.106	0.154	0.689	0.491	
adult caring	0.483	0.145	3.318	0.001	***
num. adults	0.155	0.289	0.537	0.591	
num. children	0.321	0.275	1.166	0.244	

SI Table 4: **CLM output for effect of personal circumstances on other (e.g. emotional) difficulties of self-isolation.** Household income, number of adults and number of children are log transformed. Childcare and adult caring responsibilities are binary variables. Age, household income, childcare, and adult caring responsibilities are centred.

	b	*SE*	*z*	*p*	
age	-0.033	0.005	-6.501	<0.001	***
household income	-0.457	0.188	-2.432	0.015	*
childcare	-0.122	0.155	-0.787	0.431	
adult caring	0.214	0.136	1.569	0.117	
num. adults	0.507	0.281	1.808	0.071	·
num. children	0.251	0.276	0.910	0.363	

household income (b = -0.460, SE = 0.188, z = -2.455, p = 0.014), and having adult caring responsibilities (b = 0.483, SE = 0.145, z = 3.318, p = 0.001; SI Table 3). Other difficulties were higher for younger participants (b = -0.033, SE = 0.005, z = -6.501, p < 0.001) and having a lower household income (b = -0.457, SE = 0.188, z = -2.432, p = 0.015; SI Table 4).

To investigate the role of personal circumstances on test booking, we constructed a cumulative link mixed model with the fixed effects of age (centred), household income (log transformed and centred), childcare responsibilities (centred), adult caring responsibilities (centred), number of adults in the household (log transformed), and number of children in the household (log transformed). Participant identity and trial type were included as random intercepts.

SI Table 5: **CLMM output for effect of personal circumstances on test-booking.** Household income, number of adults and number of children are log transformed. Childcare and adult caring responsibilities are binary variables. Age, household income, childcare and adult caring responsibilities are centred.

	b	*SE*	*z*	*p*	
age	0.038	0.005	6.903	<0.001	***
income	-0.714	0.208	-3.432	0.001	***
childcare	-0.146	0.173	-0.844	0.399	
adult caring	0.045	0.152	0.295	0.768	
num. adults	0.489	0.323	1.516	0.130	
num. children	0.239	0.314	0.762	0.446	

Older participants (b = 0.038, SE = 0.005, z = 6.903, p < 0.001) and participants with lower household income (b = -0.714, SE = 0.208, z = -3.432, p = 0.001) waited longer before booking a test.

2.2.3 Effect of seeing infection as unimportant

An alternative to using the perceptions of risk measures was to use the measures of seeing infection as unimportant (SI Section 2.1.1). Again, we got the same pattern of results to those we presented above. Parallel analysis of the four non-reversed items indicated a two-factor split. Factor analysis identified a factor of *community infection unimportant* — comprising the items “It doesn’t matter if people catch COVID-19” and “The consequences of catching for people are not very serious” — and a factor of *personal infection unimportant* — comprising the items “It doesn’t matter if I catch COVID-19” and “The consequences of catching COVID-19 for me are not very serious”.

For illustration purposes, mean (of individual participant means) test booking step by low and high personal and community infection unimportance is shown in SI Table 6.

SI Table 6: **Test booking step means (of individual participant means) by low and high personal and community infection unimportance, measured in test-booking steps**. Personal/Community infection unimportance is split into low (less than or equal to mean personal/community infection unimportance) and high (greater than mean personal/community infection unimportance) for illustration purposes. Our statistical analyses treat test booking step as an ordinal variable and personal and community infection unimportance as continuous variables.

			Community infection unimportance	
			Low	High
			4.85 (n = 273)	4.97 (n = 277)
Personal infection unimportance	Low	5.01 (n = 331)	4.97 (n = 213)	5.09 (n = 118)
	High	4.76 (n = 219)	4.43 (n = 60)	4.88 (n = 159)

For each preventative measure, we ran a CLMM with seeing community infection as unimportant and seeing personal infection as unimportant (both centred) as fixed effects. In the first instance, our random effects structure included random intercepts for participant identity, trial (for the test booking measure) or item (for the other four measures), age, gender, and household income (log transformed).

For the test-booking measure, seeing community infection as unimportant increased the number of steps before a participant booked a test (b = 0.243, SE = 0.100, z = 2.424, p = 0.015). There was no effect of seeing personal infection as unimportant (b = -0.125, SE = 0.082, z = -1.527, p = 0.127).

For the other preventative measures — contact tracing, between-household self-isolation intentions, within-household self-isolation intentions, and self-isolation transgression — both seeing community infection as unimportant reduced preventative measure taking ($|b| \geq 0.045$, $SE \leq 0.010$, $|z| \geq 5.159$, $p < 0.001$) and seeing personal infection as unimportant reduced preventative measure taking ($|b| \geq 0.219$, $SE \leq 0.009$, $|z| \geq 29.988$, $p < 0.001$). See SI Table 7 for details.

SI Table 7: **CLMM output for other preventative measures.** Seeing community infections as unimportant and seeing personal infections as unimportant are centred, composite measures.

	Infections unimportant	*b*	*SE*	*z*	*p*	
Contact tracing	Community	-0.385	0.010	-39.380	<0.001	***
	Personal	-0.316	0.009	-36.410	<0.001	***
Between-household self-isolation	Community	-0.421	0.009	-48.130	<0.001	***
	Personal	-0.262	0.008	-32.830	<0.001	***
Within-household self-isolation	Community	-0.045	0.009	-5.159	<0.001	***
	Personal	-0.219	0.007	-29.988	<0.001	***
Self-isolation transgression	Community	0.304	0.005	56.090	<0.001	***
	Personal	0.223	0.005	48.410	<0.001	***

3 Participant age distribution

The distribution of respondent/participant ages for Studies 1 and 2 are illustrated in SI Figure 1–2.

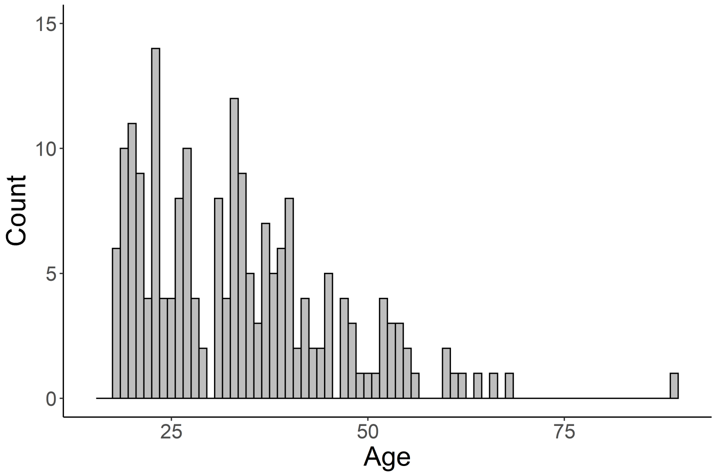

SI Figure 1: **Study 1 distribution of respondent age.** Ages ranged from 18 to 89. Note that, unlike in Study 2, we did not impose any age-related restrictions on our sample (other than that all participants were at least 18).

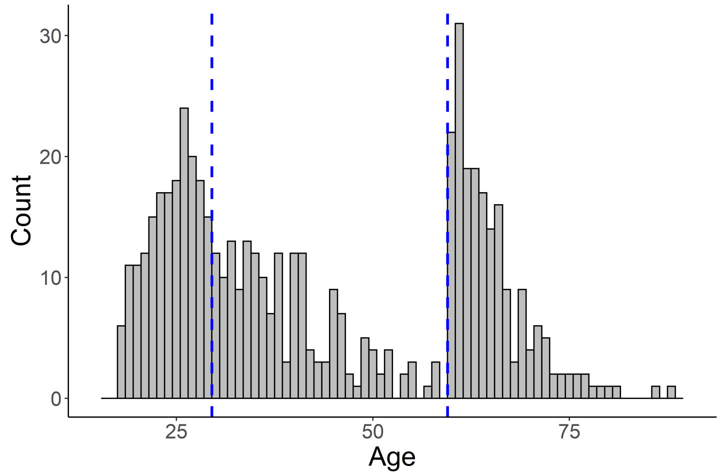

SI Figure 2: **Study 2 distribution of respondent age.** Ages ranged from 18 to 88. Vertical lines separate the three age categories used for recruitment (under 30, 30 to 59, and 60 and over).

Bibliography

Barr, D. J., Levy, R., Scheepers, C., and Tily, H. J. 2013. Random effects structure for confirmatory hypothesis testing: Keep it maximal. *Journal of Memory and Language*, 68(3):255–278.

Bates, D., Maechler, M., and Bolker, B. 2013. *lme4: Linear mixed-effects models using S4 classes.*

Christensen, R. H. B. 2019. ordinal—Regression Models for Ordinal Data.

De Vries, E. L. E. and Lee, H. C. 2022. Friend-Shield Protection From the Crowd: How Friendship Makes People Feel Invulnerable to COVID-19. *Journal of Experimental Psychology: Applied.*

Luckman, A., Zeitoun, H., Isoni, A., Loomes, G., Vlaev, I., Powdthavee, N., and Read, D. 2020. Risk compensation during COVID-19: The impact of face mask usage on social distancing. *OSF.*

R Core Team 2021. *R: A Language and Environment for Statistical Computing.* R Foundation for Statistical Computing, Vienna, Austria.
